# Relationship between Asymmetries and Functional Autonomy in Older Chilean Adults

**DOI:** 10.3390/ijerph192215063

**Published:** 2022-11-16

**Authors:** Álvaro Huerta Ojeda, Vanieska Toro-Zepeda, Emilio Jofré-Saldía, Maximiliano Bravo, Carol Parra, Gaspar Campos-Armijo, Carlos Jorquera-Aguilera, Makarena Albornoz Hernández, María-Mercedes Yeomans-Cabrera, Sergio Galdames Maliqueo

**Affiliations:** 1 Núcleo de Investigación en Salud, Actividad Física y Deporte ISAFYD, Universidad de Las Américas, Viña del Mar 2531098, Chile; 2 Facultad de Ciencias, Escuela de Nutrición y Dietética, Magíster en Nutrición para la Actividad Física y el Deporte, Universidad Mayor, Santiago 8580745, Chile; 3 Facultad de Educación y Ciencias Sociales, Instituto del Deporte y Bienestar, Universidad Andres Bello, Santiago 7550000, Chile; 4 Instituto de Ciencias de la Salud, Universidad de O’Higgins, Rancagua 2841935, Chile; 5 Servicio de Medicina Interna, Departamento de Geriatría, Hospital Carlos Van Buren, Valparaíso 2341131, Chile; 6 Laboratorio de Investigación en Nutrición y Alimentos (LINA), Departamento de Salud, Comunidad y Gestión, Facultad de Ciencias de la Salud, Universidad de Playa Ancha, Valparaíso 2340000, Chile; 7 Facultad de Ciencias, Escuela de Nutrición y Dietética, Universidad Mayor, Santiago 8580745, Chile; 8 Facultad Ciencias de la Actividad Física y del Deporte, Universidad de Playa Ancha de Ciencias de la Educación, Valparaíso 2340000, Chile; 9 Facultad de Educación, Universidad de Las Américas, Viña del Mar 2531098, Chile

**Keywords:** asymmetries, functional autonomy, Latin American Group for maturity protocol, older people

## Abstract

The objectives of this study were: (a) to determine asymmetries, both lower limb (LL) and upper limb (UL), in Chilean older adults, and (b) to relate asymmetries to FA in both LL and UL. Forty-one older adults voluntarily participated in this study (mean ± standard deviation [SD]: age 72.0 ± 8.0 years, LL asymmetries 13.78 ± 14.87%, UL asymmetries 10.70 ± 8.85%, FA 40.35 ± 16.26 points). The variables were: (1) asymmetries of LL and UL, assessed through a force platform and handgrip, respectively; (2) FA, assessed through the Latin American Group for Maturity (GDLAM) and the GDLAM index of autonomy (GI) protocol. The relationship between the variables was performed through Spearman’s correlation. The analysis showed that 39% of the participants presented asymmetries above 15% in the LL. Likewise, this 39% of older adults presented a lower FA than their peers with asymmetries below 15% in the LL (≤15%: 35.64 ± 12.26 points vs. >15%: 47.69 ± 19.23 points, *p* = 0.003). The analysis showed a small correlation between LL and GI asymmetries (*r* = 0.27, *p* = 0.07) and a small but negative correlation between UL and GI (*r* = −0.21). The mean values of asymmetries of both LL and UL are within ‘normal’ parameters. However, several older adults were identified as being at risk. In parallel, older adults who presented a higher level of asymmetries in LL showed a lower level of FA.

## 1. Introduction

By 2050, almost 2 billion people will be 60 years old or older, while eighty percent will live in developing countries [1]. In this context, it has been reported that life expectancy in Chile will be 83.2 years for men and 87.8 years for women, representing 25% of the Chilean population by 2025 [2]. In addition to these figures, the main painful conditions (such as neurodegenerative, musculoskeletal, and vascular diseases) increase incidence in older adults. In this context, D’Aiuto et al. [3] examined the associations between chronic pain conditions, pain level, and subclinical/clinical anxiety in community-dwelling older adults, reporting that older adults with three or more painful conditions have an increased risk of clinical and subclinical anxiety compared to those without do-o-full conditions. These conditions contribute to poor health-related quality of life [4], social isolation, impaired physical activity, and dependence on daily living tasks [5]. In this context, one of the major concerns for the World Health Organization is that all people in the world develop ‘healthy aging’, which is related to maintaining and improving the levels of functionality of the human being; this allows for greater physical, mental, and social well-being in the population of older adults [6].

In this line, functional autonomy (FA) corresponds to one of the most relevant health conditions in this group of people [7]. FA corresponds to the individual’s ability to perform independently and vigorously in activities of daily living [8], a factor highly associated with quality of life in older adults [9]. In this age group, it has been observed that the ability to apply strength [10,11], and with-it FA, is progressively involute with age [12,13], directly affecting activities of daily living such as household chores, gardening, or walking to a store [14]. Likewise, scientific evidence has shown that this FA decline depends on multifactorial processes involving progressive physiological deterioration [15]. This deterioration has significant consequences on older adults’ life quality, for example, sarcopenia: the degenerative and functional loss of skeletal musculature [16]. Therefore, considering this background, it is imperative to constantly evaluate FA in older adults [7,17,18,19,20]. Thus, with this diagnostic information, action plans can be created to delay the loss of function in this population [21,22].

In parallel and due to the dominance of one limb over another, it is essential to consider asymmetries between limbs as another factor associated with quality of life [23]. Specifically, asymmetries are defined as the existing differences between limbs in function and performance [24]. Although there is no consensus or prospective cohort analysis to support the criterion, most of the available literature identifies that a difference in muscle strength or power ≥ 15% between limbs in healthy subjects generates an increased risk of injury [25]. Asymmetries must be assessed according to the nature of the movements [26]. For example, because movements of the upper limbs (UL) are typically performed unilaterally, strength assessments and, thus, determination of UL asymmetries in older adults should also be performed unilaterally; in this case, the most commonly used field test is the handgrip test [27]. Following the same principle of natural movements, most motor actions of lower limbs (LL) are performed bilaterally. For this reason, the evaluation of strength and, through it, the determination of LL asymmetries in older adults should be performed bilaterally. In this case, the most used field test to evaluate the strength of LL in older adults is the Sit-to-Stand test [28]. These asymmetries can lead to body imbalances and instability, considerably increasing the risk of injuries [26] and falls in older adults [29]. Indeed, the study of asymmetries in humans has led to numerous investigations analyzing the differences between the LL and UL [23,24]. For example, it has been observed that asymmetry in hand grip strength and segmental muscle weakness is associated with a decrease in future FA [30]. Despite this, most research evaluating asymmetries has focused on adolescents [31], adults [24], and athletes [25]. However, there is evidence that a constant and early evaluation in the adult and older adult population would facilitate diagnosis and preventive intervention to reduce the incidence of asymmetries between limbs, helping to maintain FA for a more extended period in this population [32].

Based on the evidence found and described, to date, there are no studies that show the effect of asymmetries between members on FA in older Chilean adults. Consequently, the main objective of this study was to determine asymmetries, both LL and UL, in older Chilean adults. The secondary objective was to relate asymmetries to FA.

## 2. Materials and Methods

### 2.1. Research Design

**Figure 1:**
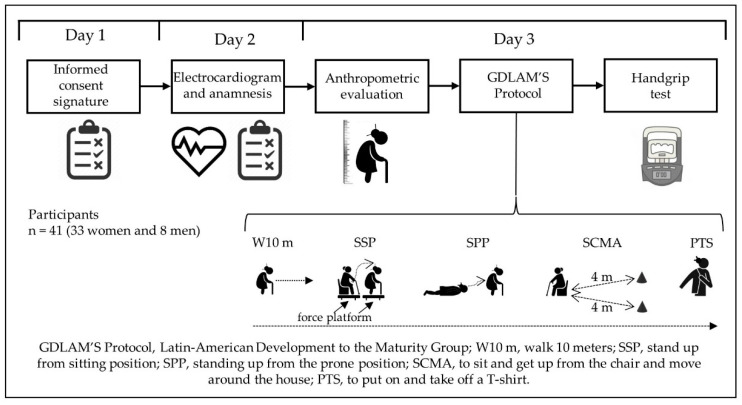
Research design.

Observational research with an associative and cross-sectional strategy. Associative, since it explores the relationships between variables to predict or explain their behavior [33] (Figure 1).

### 2.2. Participants

The sample size was calculated with a statistical program (G*Power, v3.1.9.7, Heinrich-Heine-Universität, Düsseldorf, Germany) [34]. The combination of tests used in the statistical program to calculate the sample size was as follows: (a) exact, (b) correlation: bivariate normal model, and (c) A priori: Compute required size—given α, power, and effect size. Tests considered two tails, *r* H1 = 0.45, *α*-error < 0.05, and the desired power (1-*β* error) = 0.85, slope *r* H0 = 0.00, the minimal sample size was 41 participants.

Forty-one older adults (33 women and 8 men) between 60 and 86 years of age belonging to neighborhood councils in Greater Valparaíso participated voluntarily in this study. All participants were invited to participate in the study and, in turn, recruited through the “Pedro Aguirre Cerda” Soccer Association and the “Junta de Vecinos número 17”, both from Greater Valparaíso. The evaluations were conducted between 12 July 2022 and 22 July 2022. The inclusion criteria were the following: being male or female, being 60 years of age or older and belonging to neighborhood councils in Greater Valparaíso, being able to move around autonomously and without technical assistance, having the autonomy to give consent or, if not, being represented by a family member or legal representative. In contrast, the exclusion criteria were terminal illness, uncontrolled chronic disease, severe cardiovascular conditions, severe pulmonary conditions, fractures in the last three months, neurodegenerative diseases, severe dementia, physical impossibility to perform some of the proposed tests, or refusal to sign the informed consent form. All participants were informed of the objectives of the study. Before applying the protocols, all participants signed informed consent. The study and informed consent were approved by the Ethics Committee of the Universidad de Playa Ancha, Chile (registration number: 006-2022). In addition, the study was developed under the ethical standards for exercise and sports sciences [35].

### 2.3. Anthropometric Measurements

For the characterization of the sample, weight, height, body mass index (BMI), and body-fat percentage were evaluated. Height (m) was assessed using a stadiometer from the feet to the vertex (Frankfurt plane). Weight (kg) and body fat percentage was assessed using a Tanita Inner Scan BC-554^®^ digital scale. Weight, height, and body-fat percentage were assessed with the older adults barefoot, wearing shorts, and a light T-shirt. BMI was calculated by dividing weight in kg by height in m^2^. BMI was interpreted according to the adaptation of anthropometric standards for Chilean older adults [36].

### 2.4. GDLAM’S Protocol

The Latin American Group for Maturity (GDLAM) protocol was used to assess the functional autonomy of older adults [7,17,18,37]. This protocol considers the application of five functional tests: (a) walk 10 m (W10 m), (b) stand up from the sitting position (SSP), (c) stand up from the prone position (SPP), (d) sit and get up from the chair and move around the house (SCMA), and (e) to put on and take off a T-shirt (PTS) (Figure 1).

#### 2.4.1. W10 m

The purpose of this test was to evaluate the time in seconds (s) that the participant needs to cover the distance of 10 m walking without running.

#### 2.4.2. SSP

The purpose of this test was to evaluate the functional capacity of the LL of the participants [38]. This test consists of standing up five consecutive times without assistance or resting the arms on support. The test starts from sitting, while the chair height is 50 centimeters (cm) from the floor. The unit of measurement for this test was timed in s. In addition, a force platform was used to evaluate the participants’ asymmetries during the development of this test (protocol described below).

#### 2.4.3. SPP

The purpose of this test was to evaluate the ability of the participants to rise from the floor, from the prone position with the arms at the side of the body, without assistance. The unit of measurement for this test was timed in s.

#### 2.4.4. SCMA

The purpose of this test was to evaluate the agility and balance capacity of the participants in daily life situations. With a fixed chair on the floor, with two cones diagonally to the chair (four meters to the back and three meters to the right and left sides, respectively), the participant starts the test sitting on the chair, with his feet off the floor; at the ‘go’ signal, he stands up, moves to the right, turns around the cone, returns to the chair, sits down and removes both feet from the floor; immediately, he performs the same movement to the left. This sequence is repeated twice, emphasizing completing the course in the shortest possible time. The unit of measurement for this test was timed in s.

#### 2.4.5. PTS

The purpose of this test was to evaluate the functional autonomy of the participants to dress themselves in their daily life. The test consisted of putting on a T-shirt and taking it off in the shortest possible time. The individual must be standing, with arms at the side of the body and with a large size (XL) T-shirt held in the dominant hand. On signal, the participant must put on the T-shirt and immediately take it off, returning to the initial position. The unit of measurement for this test was timed in s.

The rest interval between trials was five minutes. Then, the results of the five tests were used to calculate the GDLAM index of autonomy (GI) using the following formula [18]:

GI = [(W10 m + SSP + SPP + PTS) × 2] + SCMA]/4

### 2.5. Sit-to-Stand

During the development of the SSP test and to evaluate LL asymmetries, the force exerted in the five repetitions of the test was evaluated [28]. The force exerted by each LL during the Sit-to-Stand (STS) was evaluated through a force platform (Valkyria Trainer V1.1.8^®^, Buenos Aires, Argentina). This test was evaluated in Newton (N); this value was then divided by body mass (relative strength: N·kg^−1^ of body mass). For the statistical analysis, the mean value of the five repetitions was used with relative values: dominant leg (D), non-dominant leg (Non-D), and bilateral (BL), while the following formula was used for the calculation of asymmetry [25]:

Asymmetry = [(D − Non-D)/D] × 100

### 2.6. Handgrip Test

This test aimed to evaluate the maximum hand grip using a hand-held dynamometer (Smedley^®^ model, Tokyo, Japan) [39]. Before the evaluations, the dynamometer was adjusted to the hand size [40]. The evaluations were performed in a standing position, with the arm at the side of the body, the elbow flexing at 90°, and maintaining the maximum prehensile force for three seconds [27]. The evaluation was performed twice for both the D and Non-D hands. The rest interval between tests for the same hand was three minutes. This test was evaluated in N; this value was then divided by body mass (relative force: N·kg^−1^ of body mass). For the statistical analysis, the maximum value of the two repetitions was used for both the D and Non-D hand, while the following formula was used for the calculation of asymmetries [25]:

Asymmetry = [(D − Non-D)/D] × 100

### 2.7. Data Analysis

Data from the five functional tests of the GDLAM protocol, GI, handgrip, STS, and anthropometric parameters were sorted in a spreadsheet designed for the study. Descriptive data are presented as means and standard deviations (SD). The normal distribution of the data was confirmed by the Shapiro–Wilk test (*p* > 0.05). The ‘normal’ non-irrigated level of skewness was set at a value of <15% [25]. The correlation between variables with normal distribution was calculated using Pearson’s test, while the correlation between variables with abnormal distribution was calculated using Spearman’s test [41]. The criteria for interpreting the strength of the r coefficients were as follows: trivial (<0.1), small (0.1–0.3), moderate (0.3–0.5), high (0.5–0.7), very high (0.7–0.9), or practically perfect (>0.9). For a normal distribution, the differences in anthropometric parameters and asymmetries between men and women were calculated through the *t*-test for independent samples. In contrast, the differences in asymmetries between men and women were calculated through the *t*-test for independent samples. In contrast, the differences in asymmetries between men and women were calculated through the *t*-test for independent samples [42], for an abnormal distribution was calculated using the Wilcoxon test. The effect size (ES) was calculated with Cohen’s d test. The latter analysis considers an insignificant (*d* < 0.2), small (*d* = 0.2 to 0.6), moderate (*d* = 0.6 to 1.2), large (*d* = 1.2 to 2.0), or very large (*d* > 2.0) effect. All statistical analyses were performed with Prism version 7.00 for Windows^®^ software. The significance level for all statistical analyses was *p* < 0.05.

## 3. Results

The mean age of the older adults evaluated was 72.0 ± 8.0 years. When comparing age, weight, height, BMI, and fat percentage, only the fat percentage showed significant differences between women and men (*p* = 0.0003). The mean values of the anthropometric parameters and the comparison between sexes are reported in Table 1.

**Figure 2:**
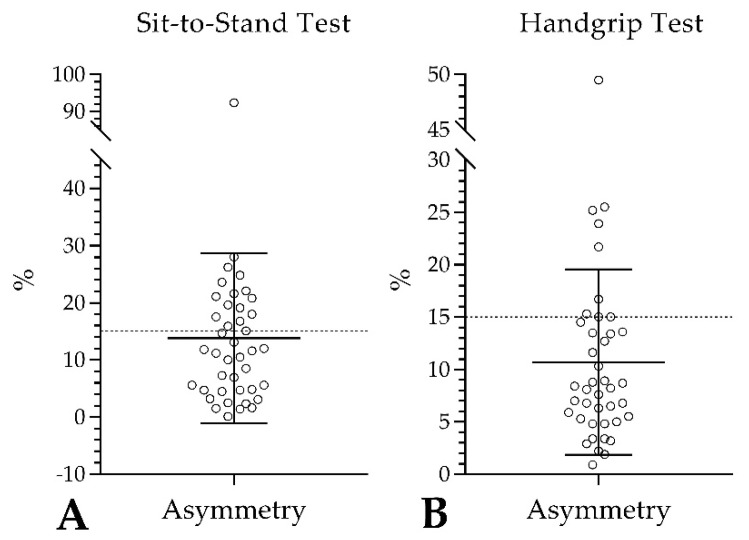
Asymmetries for the lower (**A**) and upper (**B**) limbs.

In the STS test, it was observed that the 41 older adults exerted 17.71 ± 3.55 N. When decomposing this force value, it was observed that 8.64 ± 1.80 N were performed with the D leg and 9.09 ± 1.99 N with the Non-D leg, showing significant differences between LL (*p* = 0.038). The development of strength in the STS test showed a level of asymmetry between LL equivalent to 13.78 ± 14.87%. In this same analysis, it was observed that 16 out of 41 older adults (equivalent to 39%) presented asymmetries over 15% in the LL (Figure 2A). When analyzing the STS by sex, it was observed that women exercised 17.09 ± 3.19 N, showing non-significant differences between LL (*p* > 0.05). The level of asymmetry between LL in women was 14.48 ± 16.39%. When performing the STS analysis in men, it was observed that the force exerted on LL was equivalent to 20.25 ± 4.03 N, evidencing significant differences between LL (*p* = 0.006). The level of asymmetry between LL in men was 14.18 ± 8.83%. When comparing the values of asymmetries between women and men, no significant differences were evident (women: 14.48 ± 16.39% vs. men: 14.18 ± 8.83%, *p* = 0.50). In the handgrip test, the 41 older adults exerted a force equivalent to 3.03 ± 1.06 N with the D hand and 2.93 ± 1.02 N with the Non-D hand, showing non-significant differences between UL (Wilcoxon test: *p* > 0.05). The level of asymmetry between UL was 10.70 ± 8.85%. In this same analysis, it was observed that nine out of 41 older adults (equivalent to 22%) presented asymmetries over 15% in the ULs (Figure 2B). When performing this analysis by sex, it was observed that females presented significant differences between the D and Non-D hand (D: 2.80 ± 1.01 N vs. Non-D: 2.67 ± 0.90 N, Wilcoxon test: *p* = 0.010). The level of asymmetry between UL in women was 10.80 ± 8.83%. When performing this analysis in men, non-significant differences were observed between the D and Non-D hand (D: 3.94 ± 0.76 N vs. Non-D: 4.00 ± 0.77 N, Wilcoxon test: *p* > 0.05). The level of asymmetry between UL in men was 10.30 ± 9.56%. When comparing the values of UL asymmetries between women and men, no significant differences were evident (women: 10.80 ± 8.83% vs. men: 10.30 ± 9.56%, Wilcoxon test: *p* = 0.52).

The analysis evidenced that the mean values of the five functional tests of the GDLAM (W10 m, SSP, SPP, SCMA, and PTS) and the GI classify the 41 older adults in ‘weak’ condition [34]. Specifically, of the 41 older adults assessed, 38 participants (equivalent to 92.7%) are in ‘weak’ condition, two participants (equivalent to 4.9%) are in ‘fair’ condition, and only one participant (equal to 2.4%) is in ‘good’ condition. The values of each test, the GI, and the analysis by sex are described in Table 2. In parallel, when comparing the GI between older adults presenting levels of asymmetries below and above 15% in LL (25 and 16 participants, respectively), it was evidenced that participants above 15% asymmetries among LL presented lower FA than their peers below 15% (LL asymmetries ≤ 15%: 35.64 ± 12.26 points vs. LL asymmetries > 15%: 47.69 ± 19.23 points, *p* = 0.003).

**Figure 3:**
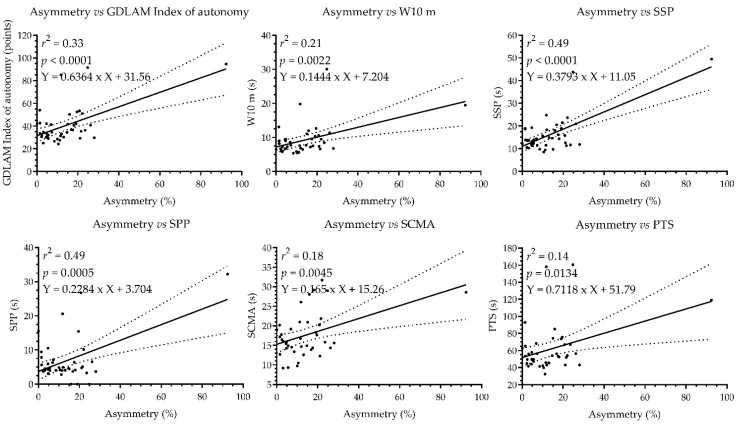
Correlation analysis between the asymmetry and GDLAM’S Protocol. W10 m, walk 10 meters; SSP, stand up from sitting position; SPP, standing up from the prone position; SCMA, to sit and get up from the chair and move around the house; PTS, to put on and take off a T-shirt; s, seconds.

When analyzing the concordance between LL and GI for the 41 older adults, we observed a small correlation (Spearman’s test: *r* = 0.27, *p* = 0.07) between asymmetry and W10 m and a moderate correlation (Spearman’s test: *r* = 0.36, *p* = 0.01), asymmetry and SSP a moderate correlation (Spearman’s test: *r* = 0.31, *p* = 0.04), asymmetry and SPP a trivial correlation (Spearman’s test: *r* = −0.07, *p* = 0.63), asymmetry and SCMA a small correlation (Spearman’s test: *r* = 0.25, *p* = 0.10), and asymmetry and PTS a small correlation (Spearman’s test: *r* = 0.22, *p* = 0.16). The correlation analysis and the respective regression lines are reported in Figure 3. Finally, when analyzing the agreement between the asymmetries of the ULs and the GI, the following were observed trivial correlations for the 41 older adults, a small correlation (Spearman’s test: *r* = −0.19, *p* = 0.21).

## 4. Discussion

In the present study, asymmetries in both LL and UL were determined in older Chilean adults. Likewise, asymmetries were related to FA, specifically to GI, both in LL and UL. Regarding asymmetry percentages, the results showed that the evaluated older adults are within ‘normal’ parameters. The results showed a small relationship between asymmetries and GI for all participants.

### 4.1. Asymmetries

After performing the STS test, 13.78 ± 14.87% of asymmetries between LL were evidenced, with significant differences between the D and Non-D leg (*p* = 0.038). In this same analysis, it was observed that 39% of the evaluated older adults present asymmetries over 15% in the LL. In this sense, there is evidence that relates asymmetries between LL with a higher risk of injury [26] and risk of falling in older adults [29]. Likewise, although there is no scientific consensus to determine the percentage of asymmetry that generates a potential risk of suffering injuries or falls, it has been identified that an asymmetry ≥ 15% corresponds to an indicator of risk in healthy subjects [25]. Indeed, observing the dispersion of our results, it is evident that several older adults with levels of asymmetries place a population at risk (16 out of 41 older adults evaluated), especially for falling [29]. However, the evidence suggests that asymmetry analyses should be performed on a case-by-case basis and consider possible modifying factors [26].

### 4.2. STS and Falling Risk

The STS evaluates the functional capacity of LL [38]. In this context, the STS can be used as a functional field test to anticipate the risk of falls in older adults [29] to clinical pathologies [32]. For example, it has been observed that patients with hip osteoarthritis have a different pattern of asymmetries compared to control patients, implying that patients with hip osteoarthritis transfer 18.4% of the total load to the unaffected leg [32]. Analyzing the concordance between LL and SSP (STS) asymmetries evaluated in the present study is interesting. This correlation revealed that older adults with higher LL asymmetries take longer to stand up (SSP or STS). Likewise, there is a possibility that older adults with a higher level of asymmetries present some clinical pathology, such as early-stage hip osteoarthritis [32]. However, the latter relationship needs further exploration.

### 4.3. Handgrip and Falling Risk

The handgrip test evaluates the maximum hand grip [39]. In this context, after evaluating hand grip in Chilean older adults, our findings evidenced a level of asymmetry between UL of 10.70 ± 8.85%, with no significant differences between the D and Non-D hand (*p* > 0.05). Regarding the asymmetries, and as mentioned in previous paragraphs, there is no scientific consensus for the ‘normal’ percentage of asymmetry [25]. Indeed, for UL asymmetries, it has been shown that the D hand tends to have 10% more strength than the non-D hand [43]. Therefore, the mean values of asymmetries observed in the present study participants can be considered ‘normal’. However, in this same analysis, it was observed that 22% of the evaluated older adults present asymmetries above 15% in the ULs. In parallel, McGrath et al. [44] showed that handgrip strength, with its respective asymmetry, predicts the risk of falls in older adults [44]. In this sense, as with the STS, the mean values of asymmetry in manual grip show a large dispersion (9 out of 41 older adults evaluated presented an asymmetry of over 15% in the ULs). This situation allows us to predict that several older adults risk falling [44]. Consequently, it is advisable to consider the calculation of UL asymmetries and thus assess the risk of falls in all protocols used with older adults.

### 4.4. Functional Autonomy in Older Adults

All activities of daily living, including interactions with people or objects, require the ability to move [45]. This independence, along with FA, is a factor directly related to the quality of life of older adults [9,45]. This study evaluated FA through GDLAM’S protocol [7,17,18,37]. In this sense, our findings showed that the 41 older adults are in a ‘weak’ condition [37]. However, based on the background described above, no national normative parameters exist to classify and qualify Chilean older adults [17]. Despite this, it has been shown that physical exercise is positively related to the maintenance of systemic functional capacity and reserve, which translates into greater FA, Independence, and quality of life in the last stage of the life cycle [46]. In this context, it is also essential to consider the personal motivations of older adults to engage in physical activity [14]. Indeed, a study by Burton et al. [14] reported that older adults prefer lifestyle activities, such as cleaning or gardening, rather than performing specific exercises. Nevertheless, it is essential to promote physical activity in older adults, as there is evidence of a positive association between different physical interventions and improvement in FA. [21]. In this sense, it is suggested that older adults perform regular physical activity, including aerobic, anaerobic, and proprioceptive exercises; in this way, they will maintain strength levels, favoring balance and reducing the risk of falls [22].

### 4.5. Asymmetries and Functional Autonomy

When analyzing LL asymmetries and their relationship with GI, it was observed that older adults with higher levels of LL asymmetries (>15%) presented less FA than their peers with levels of asymmetries ≤ 15% (*p* = 0.003). Likewise, a small correlation between the variables was observed when the asymmetries of both LL and UL were related to the FA. Despite these results, the second and third GDLAM functional tests (SSP and SPP) showed a moderate correlation between the variables. Among the five functional tests of the GDLAM protocol, some represent, to a greater degree, the FA of older adults [18,19]. This low correlation between tests could reflect that older adults naturally adapt to an increasingly adverse environment, maintaining good capacity and FA, which allows them to function in their environment and perform daily tasks. However, these antecedents need further exploration.

### 4.6. Limitations

As a first limitation of the study, it is necessary to mention that the pandemic generated by COVID-19 between 2020–2022 has caused a decrease in the participation of older adults in social activities. In this context, older adults had low acceptance of the study proposal. Among other aspects, this low number of participants did not allow us to compare different age ranges or to observe the effect of anthropometric variables (BMI) on FA. Despite this low number of participants, this first study allowed us to relate asymmetries with FA in older Chilean adults, generating an emerging line of research in a population that has become a priority in public policies in Chile.

During the study, we observed that the GDLAM qualitative scale was validated in a population different from the Chilean population [17,18,37]. Therefore, a qualitative table for the Chilean population is needed for a correct classification of GDLAM results in older Chilean adults, such as its correlation with LL and UL asymmetries, and similarly to the first limitation, in order to create this qualitative scale, a number of participants that reliably represents this population must be included.

## 5. Conclusions

The mean values of both LL and UL asymmetries are within ‘normal’ parameters and without risk for the older adults evaluated. However, it was observed that several participants have levels of asymmetries that place them in the at-risk population. In parallel, older adults who presented a higher level of asymmetries in LL showed a lower level of FA.

## 6. Prospects and Future Studies

The present research has pioneered in relating asymmetries with FA in older Chilean adults. Although a small correlation was observed between the GI, this information will help professionals and researchers who work daily with older adults to determine the autonomy profile in this population. During the development of the research, several aspects were observed that need to be addressed in future research: (a) a study is needed to determine the test–retest reliability of the GDLAM in Chilean older adults, (b) the application and evaluation of physical exercise programs, based on the individual autonomy profile, that improves FA in older adults, and (c) the creation of a qualitative table for the GDLAM that allows the qualification of Chilean older adults. Furthermore, as mentioned in the limitations of the study and to increase the representativeness of the data, it is essential to increase, in future research, the number of older adults evaluated.

## Figures and Tables

**Table 1:** Sample characterization *.

	All (*n* = 41)	Women (*n* = 33)	Men (*n* = 8)	Mean Diff	95% CI of Diff	*t*	ES	*p*-Value
Age (years)	72.0 ± 8.0	72.2 ± 8.3	71.1 ± 6.42	1.14	−7.25 to 9.54	0.354	0.15	ns
Weight (kg)	71.4 ± 14.0	70.2 ± 14.0	75.9 ± 13.7	−6.67	−14.07 to 2.72	1.752	0.41	ns
Height (m)	1.54 ± 0.08	1.51 ± 0.06	1.63 ± 0.06	−0.12	−8.52 to 8.27	0.039	1.96	ns
BMI (kg/m^2^)	30.3 ± 5.66	30.7 ± 6.02	28.0 ± 3.22	2.70	−5.69 to 11.10	0.831	0.58	ns
Fat (%)	38.0 ± 8.46	41.4 ± 6.34	27.1 ± 5.17	14.27	5.19 to 23.35	4.080	2.48	0.0003

CI, confidence intervals; diff, difference; ES, effect size; kg, kilograms; kg/m^2^, kilograms per square meter; m, meters; ns, not significant. * For the comparison between women and men, the *t*-test was used.

**Table 2:** Results of the GDLAM’S Protocol in older adults.

	W10 m (s) 95% CI of Diff	SSP (s) 95% CI of Diff	SPP (s) 95% CI of Diff	SCMA (s) 95% CI of Diff	PTS (s) 95% CI Of Diff	GI (Points) 95% CI of Diff
All (*n* = 41)	9.19 ± 4.61 7.73 to 10.65	16.29 ± 8.04 13.75 to 18.83	6.85 ± 6.58 4.77 to 8.933	17.54 ± 5.64 15.76 to 19.32	61.62 ± 27.64 52.89 to 70.34	40.35 ± 16.26 35.22 to 45.48
Women (*n* = 33)	9.67 ± 5.01 7.89 to 11.45	16.64 ± 8.67 13.56 to 19.71	7.39 ± 7.17 4.85 to 9.93	16.92 ± 5.27 15.05 to 18.79	64.74 ± 29.81 54.17 to 75.31	41.5 ± 17.64 35.25 to 47.76
Men (*n* = 8)	7.24 ± 1.11 6.30 to 8.17	14.85 ± 4.82 10.81 to 18.88	4.62 ± 2.28 2.71 to 6.53	20.07 ± 6.737 14.43 to 25.7	48.73 ± 8.07 41.98 to 55.47	35.58 ± 7.48 29.32 to 41.83

CI, confidence intervals; diff, difference; GDLAM’S Protocol, Latin-American Development to the Maturity Group; GI, GDLAM index of autonomy; W10 m, walk 10 m; SSP, stand up from sitting position; SPP, standing up from the prone position; SCMA, to sit and get up from the chair and move around the house; PTS, to put on and take off a T-shirt; s, seconds.

## Data Availability

The database of the study can be downloaded from the following link: https://doi.org/10.6084/m9.figshare.21201917 (accessed on 24 September 2022).
